# Complications and device failures associated with urolift: Findings from the MAUDE database

**DOI:** 10.1177/03915603231180016

**Published:** 2023-06-08

**Authors:** Patrick Juliebø-Jones, Bhaskar K Somani, Lazaros Tzelves, Julie Nøss Haugland, Christian Arvei Moen, Alfred Honoré, Christian Beisland

**Affiliations:** 1 Department of Urology, Haukeland University Hospital, Bergen, Norway; 2 Department of Clinical Medicine, University of Bergen, Bergen, Norway; 3 Department of Urology, University Hospital Southampton, Southampton, UK; 4 Second Department of Urology, National and Kapodistrian University of Athens, Sismanogleio General Hospital, Athens, Greece

**Keywords:** Urolift, prostate, LUTS, BPH

## Abstract

**Introduction:**

Urolift is an established intervention for symptoms of bladder outflow obstruction caused by benign prostate enlargement. Reported advantages include its minimally invasive profile, short learning curve and feasibility as a day case procedure. Our aim was to use a national registry as a means to evaluate the nature of complications and device failures that have been documented to occur.

**Methods:**

Retrospective review was performed of the US Manufacturer and User Facility Device Experience (MAUDE) database, a prospective register, which contains voluntarily submitted adverse events associated with surgical devices. Information collected include event timing, underlying cause, procedural completion, complications and mortality status.

**Results:**

Between 2016 and 2023, 103 device failures, 5 intra-operative complications and 165 post-operative complications (early: 151, late: 14) were registered. The commonest device problem (56%, *n* = 58) was failure of the implant to deploy with subsequent requirement for complete replacement. There were 50 cases of documented urosepsis. 62 patients with post operative haematuria were registered including 12 that underwent emergency embolisation. Other complications included stroke (*n* = 5), pulmonary embolism (*n* = 3) and necrotising fasciitis (*n* = 1). Twelve ITU admissions were registered. In the reports, 22 cases were filed that recorded a hospital stay of 7 days or more. Eleven deaths were captured in the database over the study period.

**Conclusion:**

While urolift is recognised as less invasive intervention compared to alternatives such as transurethral resection of the prostate, serious adverse events have been reported to occur including death. Our findings can provide learning points for surgeons and allow for improved patient counselling and treatment planning accordingly.

## Introduction

Urolift is an established surgical intervention for lower urinary tract symptoms (LUTS) caused by bladder outflow obstruction (BOO) secondary to benign prostate enlargement (BPE). Commonly reported advantages include feasibility as a day case procedure, short learning curve and a favourable complication profile in comparison to more invasive alternatives such as transurethral resection of the prostate (TURP) (Table 1).^1,2^ It also seems to demonstrate long-term durability as a re-treatment rate <15% was recently reported in a 5 year follow up study.^3^ Patient selection for urolift procedure has also been expanded as more recent studies have supported its application in patients with an indwelling urinary catheter as well as those with an obstructing median lobe.^4,5^ To this end, urolift has gained increasing popularity and achieved dissemination in clinical practice accordingly.

**Table 1.** Summary of urolift advantages and disadvantages.

Advantages	Disadvantages
• Less invasive compared to alternatives such as TURP • Short learning curve • Can be performed as day case • Can be performed under local anaesthesia • Day case procedure • No heat damage or significant tissue trauma • The implants can be easily removed if TURP performed at later date • Complications are mostly self-limiting • Preservation of sexual function • Medical therapy can be stopped • Implants can be used as surrogate markers when undergoing radiotherapy	• Limited research in large prostate burdens and obstructing median lobe • Limited research in patients with indwelling catheter • Not suitable if high bladder neck • Inferior improvement in objective outcome measures compared to alternatives such as TURP • Device failures can occur • Unknown environmental impact of single use equipment • No histology collected • Misplaced implants can develop surface encrustation • Economic advantages are lost with high number of implants use • Long term data is currently limited to one study • Radical prostatectomy at later date is more technically challenging • Negative impact on quality of later magnetic resonance imaging

TURP: transurethral resection of the prostate.

The Manufacturer and User Facility Device Experience (MAUDE) database is a prospective registry of voluntarily reported issues related to device failures and adverse events.^6,7^ It represents a relatively unique data source in that it can offer insight into issues and complications, which may fall outside of what is commonly reported in clinical studies. While it has been employed as a research tool in urology, most studies have examined events related to use of the Da Vinci robot and there has been relatively little examined in the domain of bladder outflow surgery.^8^ To this end, our aim was to review this database for events relating to urolift device in order to gain an overview of issues and complications related to its clinical use, which may be occurring.

## Materials and methods

The MAUDE database was searched for all events involving urolift since the first registration in January 2016 up until the present day (March 2023). The search term ‘urolift’ was used as well as ‘prostatic urethral lift’ and each report was individually examined. Information collected included event timing, procedural completion, complications, and mortality status. Events were categorised as either specifically relating to a potential device issue or one reporting a complication related to the urolift procedure itself. Complications were grouped as either as intra-operative or post-operative. The latter were grouped as either early (within 30 days) or late (within 12 months). Duplicate reports were excluded as well as those with insufficient information. Given all data was publicly available as well as already nonymized at source, ethical approval was not deemed necessary. Notwithstanding this, the principles of the Helsinki declaration were still upheld.

## Results

Between 1st January 2016 and 31st March 2023, our search identified 103 device failures recorded in the MAUDE database. In addition to this, five intra-operative complications and 165 post-operative complications (early complications: 151, late complications: 14) were registered.

### Device failures

The commonest problem (56%, *n* = 58) was failure of the implant to deploy with subsequent requirement for complete replacement. In 28% (*n* = 16) of these cases, more than one device failed and the maximum number of urolift devices that were ultimately required to complete an operation in a single session was six. In 71% (*n* = 41) of the cases where deployment failed, the needle was also found to be missing on inspection of the instrument afterwards. There were 22 cases of device misfire requiring instrument replacement. Other reported technical issues included fractured needle (*n* = 1), the needle becoming stuck in the prostate tissue (*n* = 1), contamination on inspection prior to use (*n* = 1), handle breakage (*n* = 1) and the filament snapping (*n* = 1).

Overall, such device problems lead to the procedure being abandoned completely in one case, while in four cases, conversion to TURP or photovaporisation of the prostate (PVP) was necessitated.

### Intra-operative complications

In total, five complications were recorded during the operation. This included four cases of sharps injury to operating staff. In one case, the needle had not retracted properly and led to injury of the primary surgeon while the other cases all related to improper handling during disposal by theatre staff. One cardiac arrest secondary to myocardial infarction was recorded. The procedure was abandoned, and the patient underwent emergency percutaneous coronary intervention (PCI) and survived.

### Post-operative complications: Early

#### Infection

There were 50 cases of documented urosepsis. Among this was a case of acute epididymo-orchitis that ultimately required orchidectomy. Two cases of urinary tract infection were recorded that were managed with oral antibiotics. There were two cases of bacterial prostatitis with one case requiring emergency TURP for abscess de-roofing. Necrotising fasciitis post urolift was registered in a single case. There were three cases of endocarditis with confirmed urinary source. Clostridium difficile (C. diff) infection was recorded in one case and was attributed to the course of oral antibiotics administered pre-operatively.

#### Haematuria

Sixty-two patients with post operative haematuria were registered on the database. While 11 patients were successfully managed with catheterisation and bladder irrigation alone, 25 required a return to theatre. Endoscopic surgery was sufficient for the mainstay of the latter, but open surgery was performed in three cases. Twelve patients were successfully managed with prostate artery embolisation but a small number ultimately required both endoscopic intervention and embolisation to gain adequate control. The maximum number of embolisation interventions required for a single patient was three. Two patients developed hyperkalaemia secondary to blood transfusions and underwent emergency dialysis accordingly.

#### Pelvic haematoma

Twelve patients were readmitted and found to have pelvic haematoma. Two of these cases were managed completely conservatively, six required blood transfusion and one patient underwent percutaneous drainage by interventional radiologist. Due to active arterial bleeding, one patient underwent embolisation while two required emergency laparotomy.

#### Migration

One case of acute bowel obstruction was recorded where emergency laparotomy was subsequently performed, and the underlying cause was determined to have been migration of an implant. Another event of migrated implant was recorded that ultimately required robotic surgery for removal, however further details were lacking.

#### Other

In addition to the patients requiring dialysis post blood transfusion, two patients were admitted with acute renal failure, of which one required emergency insertion of ureteral stent and then received dialysis in the acute setting. Three patients developed pulmonary emboli in the early post operative period and five suffered a stroke.

#### Miscellaneous

One case of nickel allergy reaction (assumed associated with nitinol implant) was registered and the patient was planned for implant removal accordingly. Two cases were reported where the patient returned within 24 h after spontaneously voiding an implant, but no further intervention was required. One procedure was found to have caused inadvertent damage to the reservoir of an inflatable penile prosthesis and this was replaced electively.

Three patients were recorded with urinary retention, while two required catheterisation alone, one did require re-operation due to the finding of a stuck needle in the prostatic urethra.

#### Intensive care admissions, deaths and hospital stay

Twelve ITU admissions were registered. In the reports, 22 cases were filed that recorded a hospital stay of 7 days or more. The longest reported stay was 6 weeks in a patient with sepsis and renal failure. Eleven deaths were captured in the database over the study period. Seven of these occurred within 30 days while the remainder occurred within 90 days post procedure. At least three of the deaths were reported to occur within the first 24 h post operatively including one case of hypovolaemic shock secondary to bleeding.

### Post-operative complications: Late

Six patients were found to have device encrustation within the first 12 months. Haematuria was the presenting complaint in half of these cases and TURP was performed in the same session as implant removal. Four patients were registered due to stricture formation post urolift. Three cases of persistent pain were found in the database that were found to have incorrectly placed implants and these cases underwent implant removal and repeat urolift or TURP. In another case of incorrect implant deployment, the patient had reported de novo urinary incontinence and underwent TURP.

## Discussion

### Key findings

This study has evaluated voluntarily reported adverse events registered in a prospective database over a 7-year period. It reveals a wide range of possible device failure issues including deployment failure and misfire. The most frequently reported post operative complication was haematuria (*n* = 62), and either return to theatre and/or embolisation was required in the majority (82%) of cases. Over the study period, 12 ITU admissions were recorded and at least 22 patients had a prolonged inpatient stay of 7 days or more. Finally, 11 mortalities were recorded in the database.

At least in the early period, urolift was promoted as a surgical intervention, which suited men who may have been younger and wanted to avoid potential sequelae of sexual dysfunction associated with alternatives such as TURP.^9^ While the nature of how events are recorded in the MAUDE database does mandate that additional information on parameters such as specific comorbidities or patient age is specified, there was a clear pattern from the event descriptions that the serious complications were seeming to occur in patients with multiple comorbidities and presumed older age. These observations would suggest that urolift is in fact also being offered as a treatment to older patients given its perceived lower anaesthetic and surgical risk profile. This is consistent with multiple ‘real world’ studies where procedural review of patient demographics reveals extreme ages including over 90 years.^10,11^

The volume of bleeding associated complications could be related to urolift being performed in larger vascular prostates. While the European Association of Urology (EAU) guidelines recommend candidates for urolift being patients with prostate burdens <70 ml, urolift surgery in patients with burdens over 100 ml and even 200 ml have been reported in the literature.^10,12^ With the additional implants required (>10) and the longer operation times including need for general anaesthesia, the truly minimally invasive profile is arguably no longer present.

A commonly reported advantage of urolift is the short learning curve,^13^ although formal studies evaluating this specific question are lacking. The array of difficulties reported in this study is perhaps a warning that the technical skills required to achieve urolift competency should not be underestimated.

The MAUDE database is a voluntary register and therefore no estimations can be made regarding incidence of reported events. It is possible that professionals may selectively choose to report those events that are more serious and unusual rather than those which occur more commonly and are more recognised in the complication profile such as acute urinary retention. While number of major complications have been reported previously such as pelvic haematoma requiring embolisation and implant placement over the ureteric orifice causing obstruction and calyceal rupture, the majority of clinical studies report serious events to occur only very rarely^10,11,14–24^ (Table 2). Moreover, several studies report no major complications to have occurred whatsoever. It seems likely that one possible explanation is that most studies are from larger centres that have already established their surgical technique. However, the reality is that urolift is being performed across a wide range of settings include outpatient clinics and community hospitals as well as general urologists who may not have a specialist interest in bladder outflow surgery. Furthermore, the anonymous nature of this database allows for serious events to be shared without the reputational impact that may occur if reported in a formal study. In 2021, Page et al. reported national data from England based on analysis of 2942 urolift procedures recorded in the Hospital Episodes Statistics database. Their findings revealed that within the first 30 days post procedure, 12% had attended the emergency department and two deaths were recorded. Interestingly, the oldest patient to undergo the procedure in that study was 97 years.^11^

**Table 2.** Summary of complications reported in the literature.

Author	Sample size	Study type	Complications (+ management if specified)	Mortality	Additional comments
McNicholas^15^	102	Multicentre retrospective	Short duration dysuria 25%, Haematuria 16% Urgency 10% Urinary retention 3% Urinary tract infection 3% Orchitis 3%	0	-
Cantwell^16^	53	Prospective multicentre crossover study	Dysuria: 36% Haematuria: 26% Pelvic pain: 21% Urgency: 8% Urinary retention: 8% Urinary tract infection: 2% Urinary incontinence 2% Bladder spasm: 2%	0	2 x CD III complications reported No CD IV or V.
Sievert^17^	68	Retrospective multicentre cohort	Transient dysuria and haematuria: 14%	0	No events > CD II
Rukstalis^18^	45	Prospective multicentre cohort	-	0	Authors state that rate of ‘serious’ complications was 0%
Kim^19^	32	Retrospective Single centre	.	0	Complications were reported as mild and transient, the most frequent being haematuria that resolved with supportive care
Pollock^20^	1	Case report	Pelvic haematoma (16.5 cm) – conservative	0	
Colemeadow^14^	1	Case report	Obstruction of Vesico-Ureteric Junction and Calyceal Rupture	0	Managed by nephrostomy, removal of the implant and resection of the median lobe
Ewing^21^	1	Case report	Pelvic haemaoma (15 cm) – blood transfusion (10 units)		
Annese^22^	35	Retrospective single centre cohort	Urinary retention (*n* = 2) Haematuria (*n* = 1)	0	Hameaturia required endoscopic intervention (CD III)
Page et al.^11^	2942	National database using Hospital Episodes Statistics	Urinary retention: 1.4%, Haematuria: 0.9% Infection: 0.1% One case with bladder injury	2 (cardiac related)	12% re-admission rate to Emergency department
Baboudijan^23^	56	Retrospective single centre	Haematuria (*n* = 1)	0	No major complications
Roehmholdt^24^	1	Case report	Pelvic haematoma (16.5 cm) – IV antibiotic therapy and transfusion	0	-
Lehner^10^	91	Retrospective single centre cohort	Haematuria: 15.4% Dysuria: 15.4% Pelvic pain: 5.5% UTI: 8.9% AUR: 3.3%	0	Re-admission to ED within 90 days: 9.9%

UTI: urinary tract infection; AUR: acute urinary retention; CD: Clavien Dindo; IV: intravenous.

## Limitations

There are limitations to acknowledge in this study. As previously mentioned, the nature of data reporting does not allow for any estimations on incidence to be generated. Moreover, patient demographics are not shared in a systematic way and details such as surgeon experience or hospital setting are lacking. However, use of this database as a research tool does offer advantages in so far as cataloguing complications that are less expected, and which occur outside of formal studies.

## Conclusions

While urolift is generally recognised as a procedure with a low morbidity profile, major complications do occur, and this can include death. The findings of this study could serve to ameliorate case selection, treatment planning and patient counselling accordingly.
